# Sirt3 modulates fatty acid oxidation and attenuates cisplatin‐induced AKI in mice

**DOI:** 10.1111/jcmm.15148

**Published:** 2020-04-12

**Authors:** Ming Li, Can‐ming Li, Zeng‐chun Ye, Jiayan Huang, Yin Li, Weiyan Lai, Hui Peng, Tan‐qi Lou

**Affiliations:** ^1^ Division of Nephrology Department of Medicine The Third Affiliated Hospital of Sun Yat‐sen University Guangzhou China

**Keywords:** acute kidney injury, cisplatin, fatty acid oxidation, mitochondrion, Sirtuin3

## Abstract

Fatty acid oxidation (FAO) dysfunction is one of the important mechanisms of renal fibrosis. Sirtuin 3 (Sirt3) has been confirmed to alleviate acute kidney injury (AKI) by improving mitochondrial function and participate in the regulation of FAO in other disease models. However, it is not clear whether Sirt3 is involved in regulating FAO to improve the prognosis of AKI induced by cisplatin. Here, using a murine model of cisplatin‐induced AKI, we revealed that there were significantly FAO dysfunction and extensive lipid deposition in the mice with AKI. Metabolomics analysis suggested reprogrammed energy metabolism and decreased ATP production. In addition, fatty acid deposition can increase reactive oxygen species (ROS) production and induce apoptosis. Our data suggested that Sirt3 deletion aggravated FAO dysfunction, resulting in increased apoptosis of kidney tissues and aggravated renal injury. The activation of Sirt3 by honokiol could improve FAO and renal function and reduced fatty acid deposition in wide‐type mice, but not Sirt3‐defective mice. We concluded that Sirt3 may regulate FAO by deacetylating liver kinase B1 and activating AMP‐activated protein kinase. Also, the activation of Sirt3 by honokiol increased ATP production as well as reduced ROS and lipid peroxidation through improving mitochondrial function. Collectively, these results provide new evidence that Sirt3 is protective against AKI. Enhancing Sirt3 to improve FAO may be a potential strategy to prevent kidney injury in the future.

## 1 INTRODUCTION

Acute kidney injury (AKI), which is an important cause of secondary chronic kidney disease (CKD) and death in hospitalized patients, has become a worldwide public health concern. Approximately 13 million people suffer from AKI every year, and approximately 1.7 million people die from AKI and its complications.^1^, ^2^ The aetiology of AKI is diverse, including severe infections, heart disease, haematological tumours and nephrotoxic drugs. Cisplatin is one of the most efficient and widely used antineoplastic in the clinic. Theapeutic effects of cisplatin are positively correlated with its dose, but so are its toxicity and side effects. Renal toxicity is one of the most common and severe reactions and greatly limits the use of cisplatin, and it was reported that the incidence rate of cisplatin‐induced nephrotoxicity is as high as 20%‐41%.^3^, ^4^

The specific mechanism of cisplatin‐induced AKI is extremely complex. Current studies suggest that cisplatin‐induced AKI is mainly related to oxidative stress, DNA damage, mitochondrial dysfunction and energy synthesis disorders, activation of the apoptotic pathway and intracellular calcium homeostasis disorders.^5^, ^6^ Meanwhile, studies have reported that there is evident fatty acid oxidation (FAO) dysfunction and lipid deposition in cisplatin‐induced AKI model.^7^, ^8^, ^9^ FAO dysfunction has also been proved an important mechanism of renal fibrosis.^10^ However, despite being the most important energy source of renal tubules, the effect of FAO dysfunction on cisplatin‐induced AKI and its specific mechanism have not yet been fully elucidated.

Sirtuin3 (Sirt3) is an important member of the sirtuin family and is the major deacetylase located in the mitochondrial matrix.^11^ Previous studies showed that Sirt3 protected against tubular injury in AKI animal model as a key regulator of mitochondrial dynamics,^12^, ^13^ which suggests that it could be a new target for improving the outcome of AKI. In addition, Sirt3 has been proved to be closely related to FAO. In a fasting mouse model, Sirt3 was found to regulate mitochondrial FAO by reversible enzyme deacetylation.^14^ However, in cisplatin‐induced AKI, whether Sirt3 participates in the regulation of FAO and thus improves the prognosis of AKI has not yet to be reported. Based on this, we constructed cisplatin‐induced AKI model in Sirt3 knockout mice to observe the effect of Sirt3 on fatty acid metabolism and further explore the possible mechanisms of these effects.

## 2 MATERIALS AND METHODS

### 2.1 Mice

All animal experiments were approved by the Sun Yat‐sen University Animal Care Committee. Sirt3 knockout (KO) mice in an SV129 background were purchased from The Jackson Laboratory, and wild‐type (WT) mice were acquired from Beijing Vital River Laboratory Animal Technology Co., Ltd. Male mice weighing approximately 20‐25 g were administered cisplatin (20 mg/kg; Sellck) or vehicle (saline) by a single ip injection as previously reported.^15^, ^16^ To investigate the effects of honokiol (HKL) on mice with cisplatin‐induced AKI, the mice were pre‐treated with 5 mg/kg HKL dissolved in dimethyl sulfoxide (DMSO) per day by i.p. for 3 days before cisplatin injection. After 72 hours of cisplatin injection, the mice were anesthetized and executed. Blood was taken from the eyeball for biochemical examination, and kidney tissue was taken for pathological examination.

### 2.2 Cell culture

Mouse renal tubular epithelial cells were obtained from the American Type Culture Collection and cultured in DMEM‐F12 medium containing 10% foetal bovine serum (Invitrogen), 100 U/mL penicillin and 100 mg/mL streptomycin in a 5% CO_2_/95% air atmosphere at 37°C. When the cells reached 80%‐90% confluence, they were stimulated with cisplatin (5 µmol/L) in the presence or absence of HKL (5 µmol/L). After 24 hours, the cells were collected for further experiments.

### 2.3 Analysis of renal function

Renal function was evaluated by measuring serum creatinine (Scr) and blood urea nitrogen (BUN). A Serum Creatinine Colorimetric Assay Kit (Cat #700460, Cayman Biochemical) was used to detect Scr levels. And BUN level was measured by BUN assay kits (Cat# C013‐2‐1, Jiancheng Biotech). All procedures were performed according to the kit's instructions.

### 2.4 Histology and immunohistochemistry

Kidney tissues were fixed in 4% paraformaldehyde. Deparaffinized sections were stained with Periodic acid‐Schiff (PAS) staining and observed by light microscopy. A score was assigned according to the percentage of tissue in the corticomedullary region exhibiting renal tubulointerstitial injury per 10 high‐power fields as follows: 0 = 0%, 1 = <10%, 2 = 10%‐25%, 3 = 25%‐50%, 4 = 50%‐75%, 5 = more than 75%, as previously described.^17^

For 4‐hydroxy‐2‐nonenal (4HNE) and phosphorylated AMPKα immunohistochemical staining, 5‐mm kidney sections were incubated with anti‐4‐hydroxynonenal antibody (1:50, Cat# MAB3249‐SP, R&D Systems) and anti‐phospho‐AMPKα (THR 172) rabbit mAb (1:100, Cat# 50081, CST), followed by incubation with the relevant peroxidase‐conjugated secondary antibodies. Positive tubular regions in the renal corticomedullary area were quantified in 10 random visual fields (200×) per kidney section.

### 2.5 Oil red O staining

The frozen sections of kidney were washed with PBS three times, then heated at room temperature and soaked in distilled water, after which the embedding agent was washed out. The sections were immersed in 60% isopropanol for 2 minutes, dyed with oil red O solution for 30 minutes at room temperature, rinsed with double distilled water 3 times and then re‐dyed with haematoxylin for 1‐2 minutes. The glass was then rinsed in running water for at least 5 minutes and dried, and the glycerine gelatin‐mounted sections were observed and photographed under a microscope.

### 2.6 Measurement of free fatty acid (FFA) content

A non‐esterified fatty acid (NEFA) test kit (KeyGen Biotech) was used to detect the FFA content. All procedures were performed according to the kit's instructions.

### 2.7 Quantitative analysis of energy metabolites in kidney tissue by LC‐MS

Kidney tissues ground in liquid nitrogen were extracted by methanol/acetonitrile (1:1, v/v) with low‐temperature ultrasound, and then, the proteins were precipitated for 1 hour at −20°C and centrifuged at 13 000 *g* at 4°C for 15 minutes to obtain the supernatant. The samples were separated by an Agilent 1290 Infinity LC system (Agilent Technologies) equipped with a 2.1 mm × 100 mm column (Agilent XDB C18). The column temperature was maintained at 45°C, and the flow rate was 0.3 mL/min with a gradient over a 23 minutes run. A gradient was run starting with 60% buffer A containing ammonia acetate and 40% buffer B containing acetonitrile and ending with 90% B; 40% B was used as the column buffer for 0‐18 minutes, which was increased to 90% B during 18‐18.1 minutes, following which buffer containing 90% B was held for 18.1‐23 minutes. To detect and evaluate the stability and repeatability of the system, QC samples were set at intervals. A 5500 QTRAP mass spectrometer was used for MS analysis (negative ion mode). The original MRM data from the energy metabolites were extracted by Analyst software, and the peak area of each metabolite was obtained.

### 2.8 mRNA preparation and quantitative real‐time RT‐PCR

Total RNA was extracted from mouse kidney with a TRIzol reagent (Invitrogen) and precipitated in isopropanol. Then, a PrimeScript™ RT Reagent Kit with gDNA Eraser (Takara) was used for the total RNA reverse transcription. SYBRR Premix Ex Taq™ II (Takara) was used to amplify cDNA. The expression levels of the target genes were normalized by β‐actin expression.

The sequences of the primers are listed as follows: SIRT3 (mouse): forward, CACGTTTACAAACATGAACC, reverse, CATGCTAGATTGCCCTAGT; β‐actin (mouse):forward, AGACCTTCAACACCCCAG, reverse, CACGATTTCCCTCTCAGC.

### 2.9 Western blot and immunoprecipitation

Proteins from the kidney cortex and kidney cells were extracted in RIPA lysis buffer. After protein concentrations were determined by the BCA method, the samples were adjusted to the same protein concentration with pyrolysis solution. The protein samples were mixed with 4× loading buffer at a 1:3 ratio and boiled for 5 minutes at 100°C. According to the content of the target protein, samples containing equal amounts of protein were electrophoresed through 10% SDS‐PAGE gels and transferred to PVDF membranes (Millipore Corp.) at 100 V. BSA/TBST (5%) was used to block non‐specific binding sites, and the membranes were subsequently incubated overnight at 4°C with the following primary antibodies: monoclonal rabbit anti‐ Sirt3 antibody (1:1000, Cat# 5490,CST), CPT1A antibody (1:1000, Cat# 15184‐1‐AP, Proteintech), PPARα antibody (1:1000, Cat# 15540‐1‐AP, Proteintech), monoclonal rabbit anti‐ACADL/LCAD (1:1000,Cat# ab196655, Abcam), Total OXPHOS Rodent WB Antibody Cocktail (Cat# ab110413, Abcam), phospho‐AMPKα (Thr172) (D4D6D) rabbit mAb (1:1000,Cat# 50081, CST), AMPKα (D63G4) rabbit mAb (1:1000, Cat# 5832, CST), cleaved caspase‐3 rabbit mAb (1:1000, Cat# 9664, CST), anti‐acetyl coenzyme A carboxylase (1:2000, Cat# ab45174, Abcam), and anti‐acetyl coenzyme A carboxylase (phospho S79) antibody (1:5000, Cat# ab68191, Abcam), GAPDH antibody(1:5000, Cat# 10494‐1‐AP, Proteintech). After being washed three times, the membranes were incubated with a horseradish peroxidase (HRP)‐labelled goat anti‐rabbit antibody or goat antimouse antibody (1:20 000, Bioworld Technology) for 1 hour at room temperature in 5% BSA/TBST. The membranes were washed three times. Protein bands were detected by enhanced chemiluminescence (ECL, Millipore) and quantitatively analysed by normalizing to the level of GAPDH using ImageJ software (NIH).

According to the manufacturer's instructions, 100 µL renal tissue protein samples was added to 1 µg Anti‐liver kinase B1 (LKB1) antibody (Cat# 10746‐1‐AP, Proteintech) and incubated on a rocking platform overnight at 4°C. Then, 20 µg Protein AG Agarose Breads (Cat# 10600‐P07E‐RN; Sino Biological) was used to capture the conjugated polymers at 4°C for 4 hours. Immunoprecipitates were collected by centrifugation at 3000 *g *for 5 minutes at 4°C and washed with uptake buffer for four times. Proteins bound on beads were analysed by Western blot using acetyl‐lysine antibody (1:1000).

### 2.10 ACADL activity assay

ACADL activity of renal tissue lysate was determined by colorimetry using LCAD activity assay kit (Genmed Science). Palmitoyl coenzyme A was used as a substrate through the coupling reaction of phenazine methoxysulfate (PMS)—2,6‐dichlorophenol.

### 2.11 Apoptosis assay

An annexin‐FITC apoptosis detection kit (BD Biosciences) was used to detect apoptosis by flow cytometry using cells at a concentration of 1 × 10^6^cells/mL. Cells were cultured and treated as indicated. Cells were resuspended in binding buffer, stained with annexin V‐FITC/PI (annexin V‐FITC: PI, 1:2) according to the manufacturer's instructions and incubated at room temperature for 15 minutes in the dark. Then, the cells were detected by flow cytometry. The number of apoptotic mRTECs was the sum of early apoptotic cells AV (+) and PI (−) plus late apoptotic cells AV (+) and PI (+).

Kidney tissue apoptosis was measured using terminal deoxynucleotidyl transferase‐mediated dUTP nick‐end labelling (TUNEL) staining (Roche Molecular Biochemicals). Ten randomly selected microscopic fields magnified at 200× were examined for each section, and the percentage of TUNEL‐positive cells was calculated in different mice.

### 2.12 Transmission electron microscopy (TEM)

The upper polar cortex of each mouse kidney was removed and immobilized in 2.5% neutral glutaraldehyde phosphate buffer for more than 24 hours at 4°C. The kidney tissue was immobilized with 1% osmium acid (1 g osmium crystal dissolved in 0.1 mol/L phosphate buffer) for 2 hours. The tissue was rinsed 3 times with 0.1 mol/L phosphate buffer for 10 minutes each time. All the above processes were carried out at 4°C. Then, the tissues were immersed in different concentrations of acetone for dehydration. An epoxy resin mixture/pure acetone (1:1) was added, and the tissues soaked for 24 hours before being embedded in an incubator at 60°C for 24 hours. An ultramicrotome was used to cut the slices to a thickness of approximately 500 A. Then, the slices were double stained with uranium acetate and lead nitrate and observed under a JEM‐1400 transmission electron microscope. Five fields were randomly photographed at the same magnification, and approximately 20 mitochondria in each field were randomly selected for analysis. The aspect ratio was calculated as previous published methods.^18^

### 2.13 Measurement of ATP production

An enhanced ATP detection kit (S0026; Beyotime) was used to determine ATP production. A total of 100 mL of pyrolysis solution was added to every 10 mg of renal tissue, which was then fully homogenized with a glass homogenizer. After pyrolysis, the tissue lysate was centrifuged for 10 minutes at 4°C and 12 000 *g*, and the supernatant was collected for testing. An ATP standard was diluted in ATP pyrolysis solution to an appropriate set of concentrations according to the manufacturer's instructions, and a standard curve was produced. Before ATP detection, 100 µL ATP detection solution was added to each test tube for 5 minutes at room temperature. Then, 20 µL of standard or sample was added to each tube and mixed quickly. The RLU values were read by a luminometer. The concentration of ATP in each sample was calculated according to the luminescence signal and normalized by protein concentration.

### 2.14 Assay of ROS level

Reactive oxygen species (ROS) levels in kidney tissues were detected by DCFH‐DA (Beyotime) test kit. In brief, tissue homogenates were diluted 1:20 in cold Locke's buffer to obtain a concentration of 5 mg tissue/mL. The reaction mixture containing the homogenate and DCFH‐DA (5 mmol/L) was incubated for 15 minutes at room temperature. After 30 minutes of further incubation, the conversion of DCFH‐DA to the fluorescent product DCF was measured by a confocal laser scanning microscope with excitation at 488 nm and an emission at 525 nm. ROS formation was quantified using a DCF‐standard curve, and data were expressed as pmol DCF/min/mg protein.

Reactive oxygen species in the mitochondria were stained with MitoSOX Red mitochondrial superoxide indicator (Invitrogen). mRTECs were seeded on a special dish for laser confocal focusing. A working solution of 5 µmol/L MitoSOX was prepared in HBSS. The working solution was added to the dish and incubated in a 37°C cell incubator for 10 minutes. HBSS was used to wash off the staining solution 3 times, and then, the nuclei were stained with DAPI for 15 minutes. After cleaning, the cells were detected by laser confocal microscopy.

### 2.15 Statistical analysis

Statistical analysis was performed using the statistical package SPSS for Windows version 13.0 (IBM SPSS) and GraphPad Prism 6.0 (GraphPad Software). All data are presented as the means ± SEMs. Statistical comparisons of the groups were performed using the *t* test or one‐way analysis of variance (ANOVA), and *P* < .05 indicated statistical significance in all tests.

## 3 RESULTS

### 3.1 Lipid accumulation and fatty acid oxidation dysfunction in mice with cisplatin‐induced AKI

**Figure 1:**
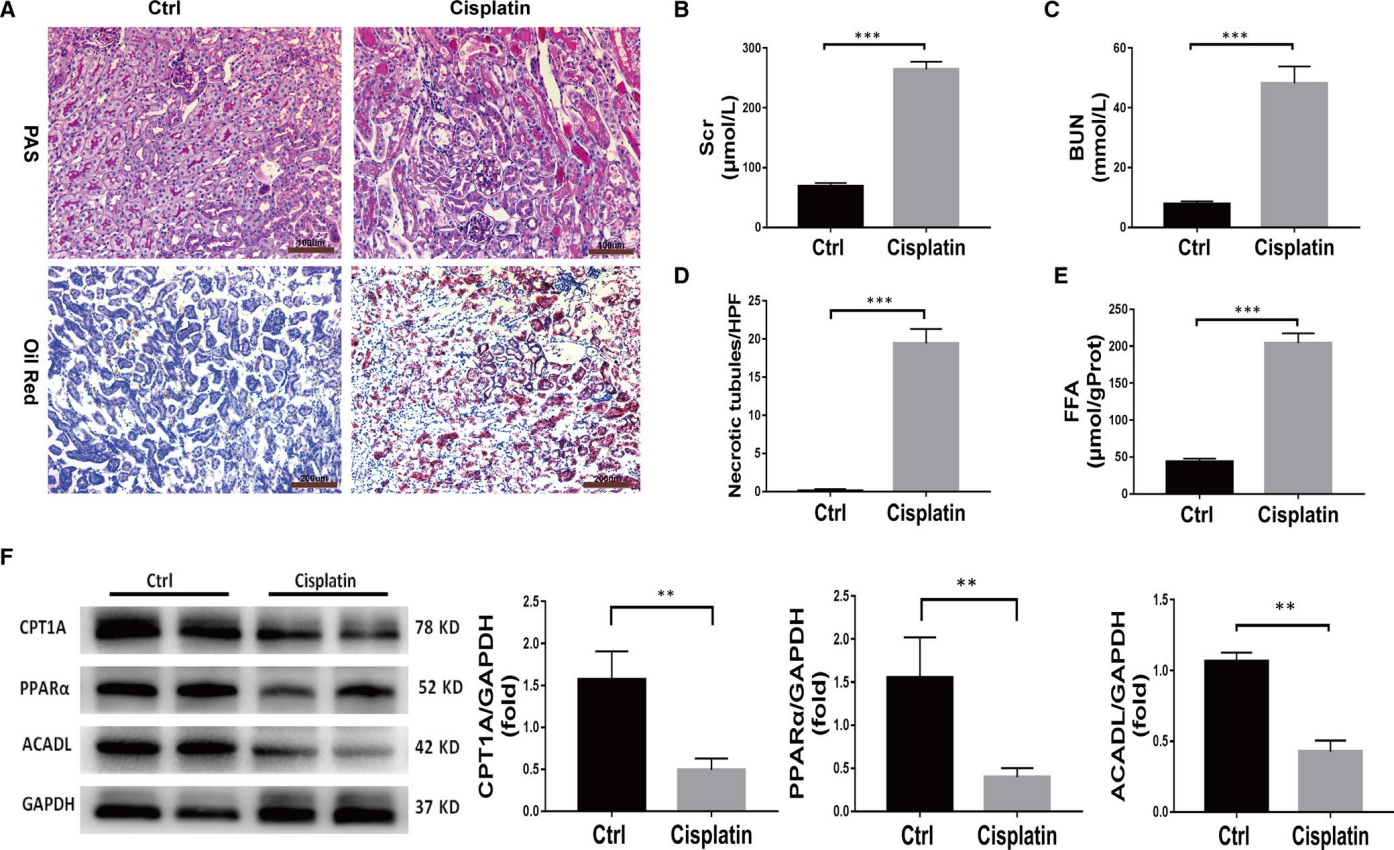
Mice with cisplatin‐induced AKI exhibit fatty acid oxidation dysfunction and lipid deposition. A, PAS staining (scale bar, 100 µm) and oil red O staining (scale bar, 200 µm) of kidney tissue from wild‐type (WT) mice in the control group and the cisplatin group. B, Comparison of serum creatinine (Scr) levels between the control and cisplatin‐treated WT mice, n = 6, ***P < .001. C, Comparison of blood urea nitrogen (BUN) levels between the control and cisplatin‐treated WT mice, n = 6, ***P < .001. D, Quantitative analysis of necrotic tubules. Data are the mean ± SD of 20 random fields from each kidney, ***P < .001, n = 3. E, Comparison of the FFA concentrations in the control and cisplatin‐treated WT mice, n = 6, ***P < .001. F, Western blot and densitometric analysis of key fatty acid oxidation proteins in the kidney tissue of mice in the control group and cisplatin group, n = 4, **P < .01

Seventy‐two hours after cisplatin exposure, the mice developed AKI characterized by proximal tubular injury (Figure 1A,D). The Scr and BUN levels were significantly higher in cisplatin‐treated mice than in control mice (Figure 1B,C). Oil red staining showed evident lipid accumulation in the kidneys of cisplatin‐treated mice, while no lipid accumulation was found in the control mice (Figure 1A). Consistent with the change in pathological morphology, the FFA level, which was detected in kidney tissue, was higher in cisplatin‐treated mice than in control mice (Figure 1E). In addition, the expression of FAO‐related key proteins in cisplatin‐induced AKI mice was detected by Western blotting. In line with lipid accumulation, the levels of PPARα, CPT1A and ACADL in mice with cisplatin‐induced AKI were significantly lower than those in control mice (Figure 1F).

### 3.2 FAO dysfunction and lipid accumulation lead to metabolic disorders and FFA‐related lipotoxicity, and thus mRTECs apoptosis

**Figure 2:**
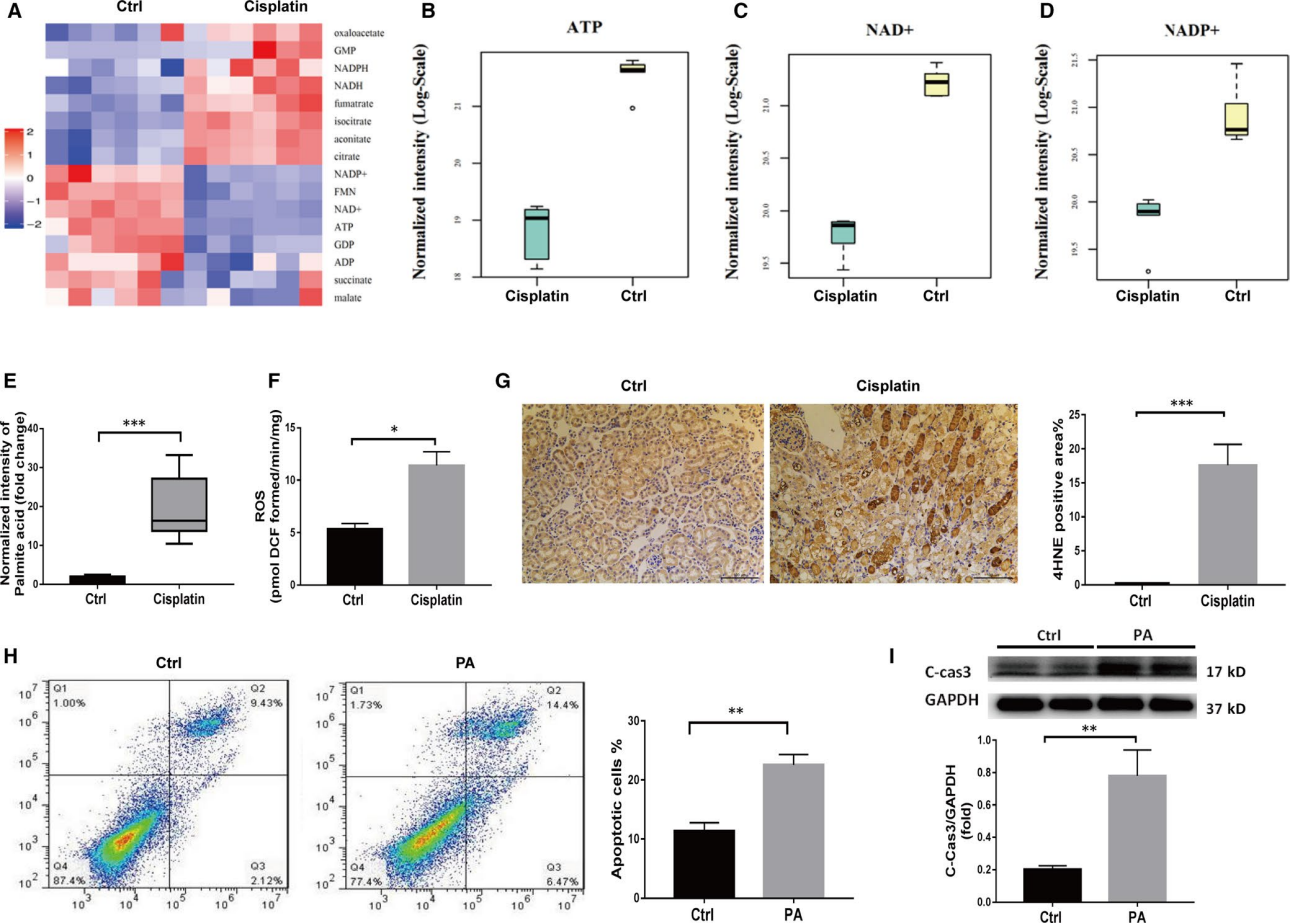
Metabolic disorders and FFA‐related lipotoxicity increase the apoptosis of mRTEcs. A, Metabolomics analysis of the kidney cortex in the control group and the cisplatin group. Heat map showing the relative levels of metabolites in kidneys from mice in each group (n = 6). B‐D, LC‐MS detection of ATP, NAD+, NADP+ in the kidney cortex of mice in the control group and the cisplatin group, n = 6, *P < .05. E, LC‐MS detection of palmitic acid level in the kidney of mice in the control group and the cisplatin group, n = 6, ***P < .001. F, Comparison of ROS between the control and cisplatin‐treated WT mice, n = 3, *P < .05. G, Representative 4HNE immunohistochemical staining of mouse kidney sections from the control group and the cisplatin group and the semiquantitative positive 4HNE staining scores, scale bar, 100 µm. H, The apoptotic rates of mRTEcs in the control group and the palmitic acid group were detected by V‐FITC/PI staining, **P < .01, n = 3. I, Western blot and densitometric analysis of cleaved caspase‐3 in mRTEcs in the control group and the palmitic acid group, **P < .01, n = 4

To further explore the effect of FAO on mice with cisplatin‐induced AKI, metabolomics of tricarboxylic acid (TCA) cycle and FFA‐related lipotoxicity were investigated. Metabolomics analysis of TCA cycle revealed that there were significant energy utilization obstacles in the kidneys from mice with cisplatin‐induced AKI. The levels of ATP, ADP, NAD+, NADP+, FMN and other TCA products were significantly lower than those in the control mice (Figure 2A‐D). As FAO is the main fuel for renal tubular epithelial cells, it is believed that impaired FAO greatly affects energy metabolism in renal tubules, as well as the regeneration of these tubules. Recently, free fatty acid (FFA) mediated lipotoxicity received attention as a leading cause of tubulointerstitial damage.^19^, ^20^, ^21^ Among several kinds of FFAs, saturated FFAs such as palmitate or palmitic acid (PA) are more toxic than unsaturated FFAs. The accumulation of PA increases the ROS of mitochondria and induces inflammation and apoptosis. In addition, the accumulation of PA‐mediated diacylglycerol contributes to the activation of protein kinase C isoforms, followed by tissue impairments.^21^, ^22^ With the help of mass spectrometry analysis, we found that the concentration of PA in the cisplatin group was 19 times higher than in the control group (Figure 2E). Base on this, to simulate the lipotoxicity of fatty acid accumulation, mRTECs were incubated with palmitic acid (0.2 mmol/L) for 24 hours. Flow cytometry was used to detect the apoptosis of mRTECs. Compared to the control group, the apoptosis rate of mRTECs in the PA group was significantly higher (Figure 2H). Consistent with flow cytometry, WB analysis showed that the expression of cleaved caspase‐3 in PA group was significantly higher than that in control group (Figure 2I). In vivo, it was found that the ROS in kidney tissue of cisplatin group was significantly higher than that of the control group (Figure 2F). Meanwhile, it was shown that the expression of 4HNE in the mouse kidney, a marker of lipid peroxidation, was increased with cisplatin treatment (Figure 2G), suggesting that lipid accumulation could induce lipid peroxidation. At present, lipid‐induced cell dysfunction is also believed to be closely related to ferroptosis, while the latter is associated with lipid peroxidation and characterized by the loss glutathione peroxidase 4 (Gpx4) activity, a key enzyme for plasma membrane repair.^23^, ^24^ Here, our result also shows that Gpx4 expression was significantly decreased in mice with cisplatin‐induced AKI compared to that in control mice (Figure S1).

### 3.3 Sirt3 knockout aggravates FAO dysfunction and kidney damage in mice with AKI

**Figure 3:**
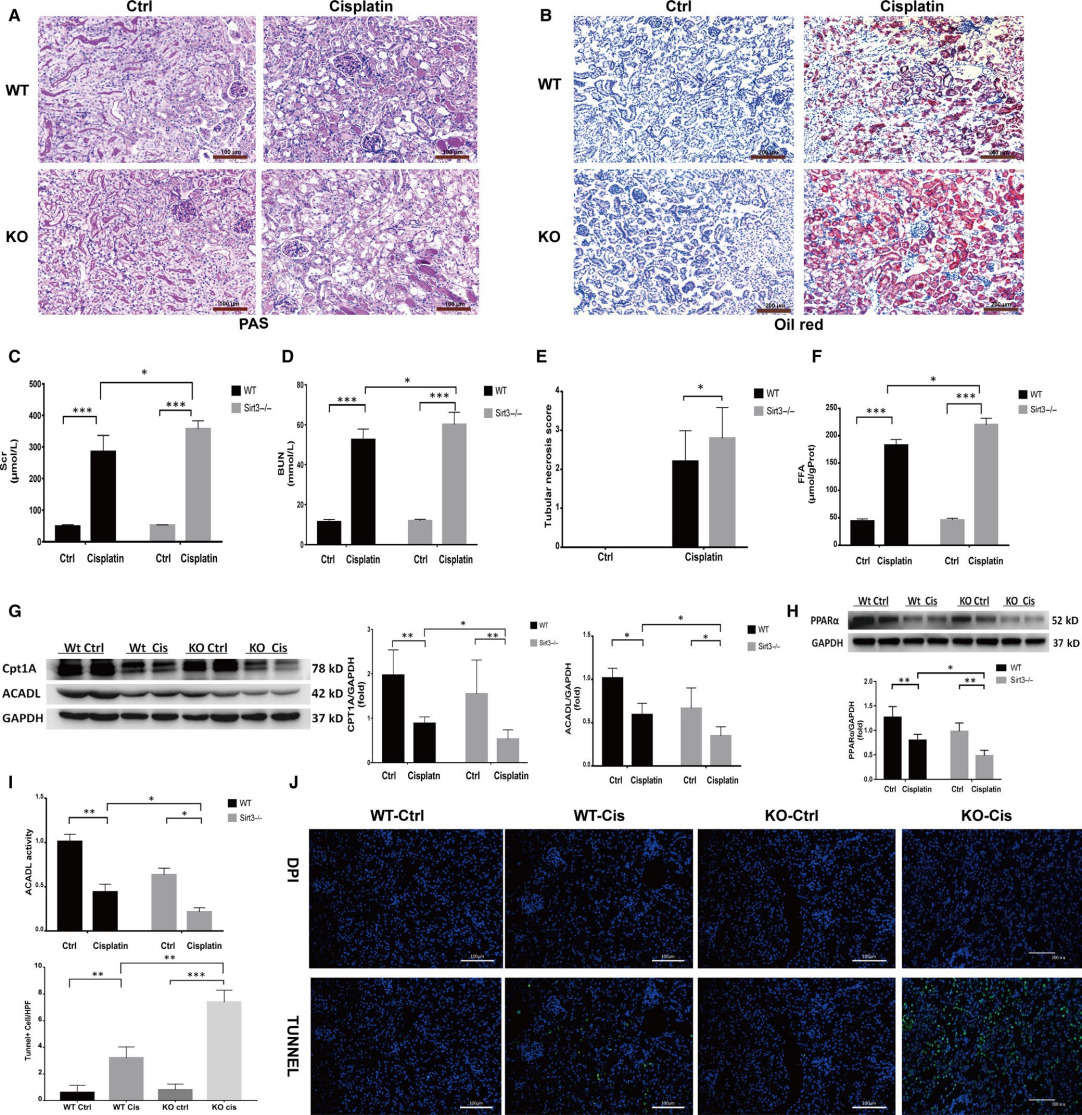
Sirt3 knockout aggravates FAO dysfunction and kidney damage in mice with AKI. A, Pathological changes in kidney tissue sections from mice in each group, as shown by PAS staining, scale bar, 100 µm. B, Comparison of oil red O staining in kidney tissue sections from mice in different groups, scale bar, 200 µm. C, Comparison of serum creatinine levels in mice in each group, *P < .05, ***P < .001, n = 6. D, Comparison of blood urea nitrogen (BUN) levels in mice in each group, *P < .05, ***P < .001, n = 6. E, Quantitative comparison of the renal tubular necrosis score for each group with 20 HP field of vision, *P < .05, n = 3. F, Comparison of the FFA concentrations in kidney tissues from mice in each group, *P < .05, ***P < .001, n = 6. G, Western blot and densitometric analysis of CPT1A, ACADL in mice in different groups, *P < .05, **P < .01, n = 4. H, Western blot and densitometric analysis of PPARα in mice in different groups, *P < .05, **P < .01, n = 4. I, Comparison of ACADL activity in mice in each group, *P < .05, **P < .01, n = 4; J, TUNEL staining of kidney tissues from mice of each group, scale bar, 100 µm. Compared with TUNEL‐positive cells in each group, n = 10 HP visual field, **P < .01, ***P < .001

Recent studies have indicated that Sirt3 is a key mitochondrial protein that prevents AKI damage^12^, ^13^, ^25^ and may be involved in FAO. As previously reported, PCR and Western blotting revealed that the mRNA and protein expression of Sirt3 in mice with cisplatin‐induced AKI was significantly lower than that in the control group. To further elucidate the role of Sirt3, we constructed cisplatin‐induced AKI model in Sirt3 knockout (KO) mice. In these mice, cisplatin was found to induce remarkably severe renal dysfunction when compared to WT mice. The Scr and BUN levels were significantly higher in KO AKI mice than in WT AKI mice (Figure 3C,D). PAS staining showed that renal tubular necrosis was more severe in KO AKI mice (Figure 3A,E). Consistently, oil red staining showed that lipid accumulation in KO AKI mice was significantly higher than that in WT mice (Figure 3B). Further, higher levels of FFA were detected in the kidney tissue of the KO AKI mice than in WT AKI mice (Figure 3F). We also used TUNEL staining to detect renal apoptosis in vivo, as shown in Figure 3J. Compared to WT AKI mice, the number of apoptotic cells in the Sirt3 KO AKI mice significantly increased. These results indicate that Sirt3 may have a protective effect against cisplatin‐induced AKI by improving fatty acid metabolism. Thus, the expression of FAO‐related proteins was examined. The results revealed that the expression of PPARα, CPT1A and ACADL was significantly lower in Sirt3 KO mice with AKI than in the WT group (Figure 3G,H). Given that ACADL is an important dehydrogenase in FAO, we further investigated the activity of ACADL and found that ACADL activity of Sirt3 KO mice was significantly lower than that of WT mice (Figure 3I). Taken together, these results suggest that Sirt3 knockout may result in decreased in FAO protein levels and activity, which aggravates FAO disorder and FFA accumulation and thus subsequently aggravates renal damage.

### 3.4 The up‐regulation of Sirt3 expression by HKL improves cisplatin‐induced AKI by restoring FAO and reducing lipid accumulation

**Figure 4:**
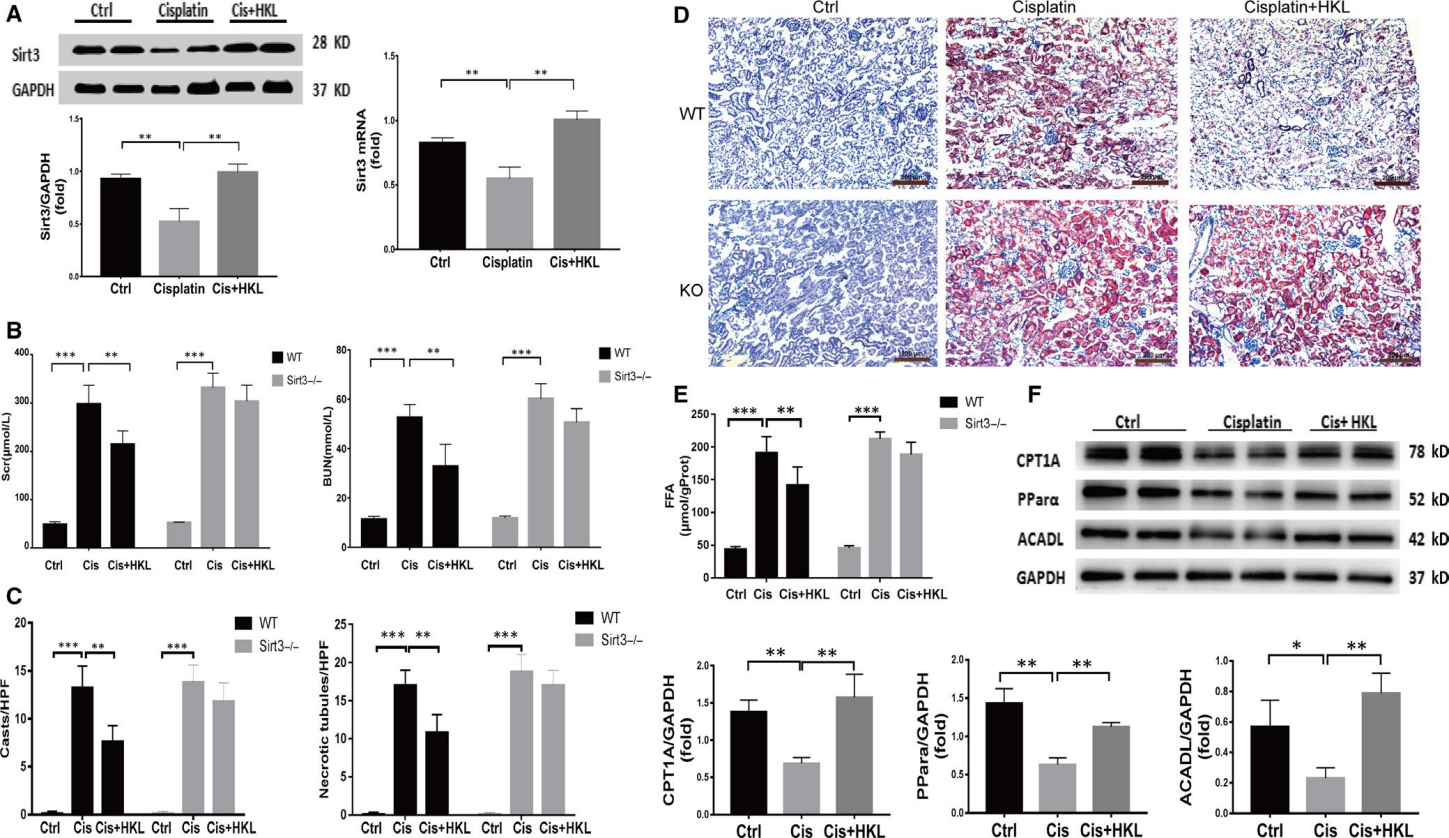
The up‐regulation of Sirt3 expression by HKL improves FAO dysfunction and renal function. A, Western blot (n = 4) and real‐time PCR analysis (n = 3) of Sirt3 in the renal tissue of control and cisplatin‐treated mice after saline or HKL administration, **P < .01. B, Comparison of renal function evaluated as serum creatinine and blood urea nitrogen in mice in each group, ***P < .001, **P < .01, n = 6. C, Comparison of renal histological changes evaluated as number of cast and necrotic tubules per HPF. ***P < .001, **P < .01. D, Oil red O staining showed lipid deposition in the kidneys of mice in each group, scale bar, 200 µm. E, Comparison of the FFA concentrations in kidneys from mice in each group, ***P < .001, n = 6. F, Western blot and densitometric analysis of key proteins related to FAO in the kidneys of mice in each group, **P < .01, ***P < .001, n = 4

Given that HKL is recognized as a Sirt3 agonist,^26^, ^27^, ^28^ it was reported HKL can attenuate cisplatin‐induced acute cytotoxicity in renal epithelial cells via cellular oxidative stress and cytoskeleton modulations.^29^ Next, we evaluated whether HKL protected mice from cisplatin‐induced AKI via improving FAO dysfunction. PCR and Western blotting revealed that mRNA and protein expression of Sirt3 was markedly increased in the mice treated with cisplatin + HKL than in the cisplatin‐treated group (Figure 4A). Parallel to this, in the WT AKI mice, pre‐treatment with HKL also improved renal function, evaluated as Scr and BUN (Figure 4B) and ameliorated tubular injury in terms of reduction of hyaline casts and tubular cell necrosis (Figure 4C). The protective effect of HKL was associated with reduced FFA accumulation. Oil red staining showed that lipid accumulation was significantly reduced and the concentration of FFA in kidney tissues significantly decreased after HKL pre‐treatment in WT mice (Figure 4D,E). However, similar results were not observed in Sirt3 KO mice. These results suggest that HKL can improve FAO and reduce FFA accumulation by up‐regulating Sirt3 expression, thus improving renal function. Further, Western blotting results showed that HKL up‐regulates the FAO‐related key proteins PPARα, CPT1A and ACADL (Figure 4F).

### 3.5 Sirt3 may regulates FAO Via the LKB1‐AMPK pathway

**Figure 5:**
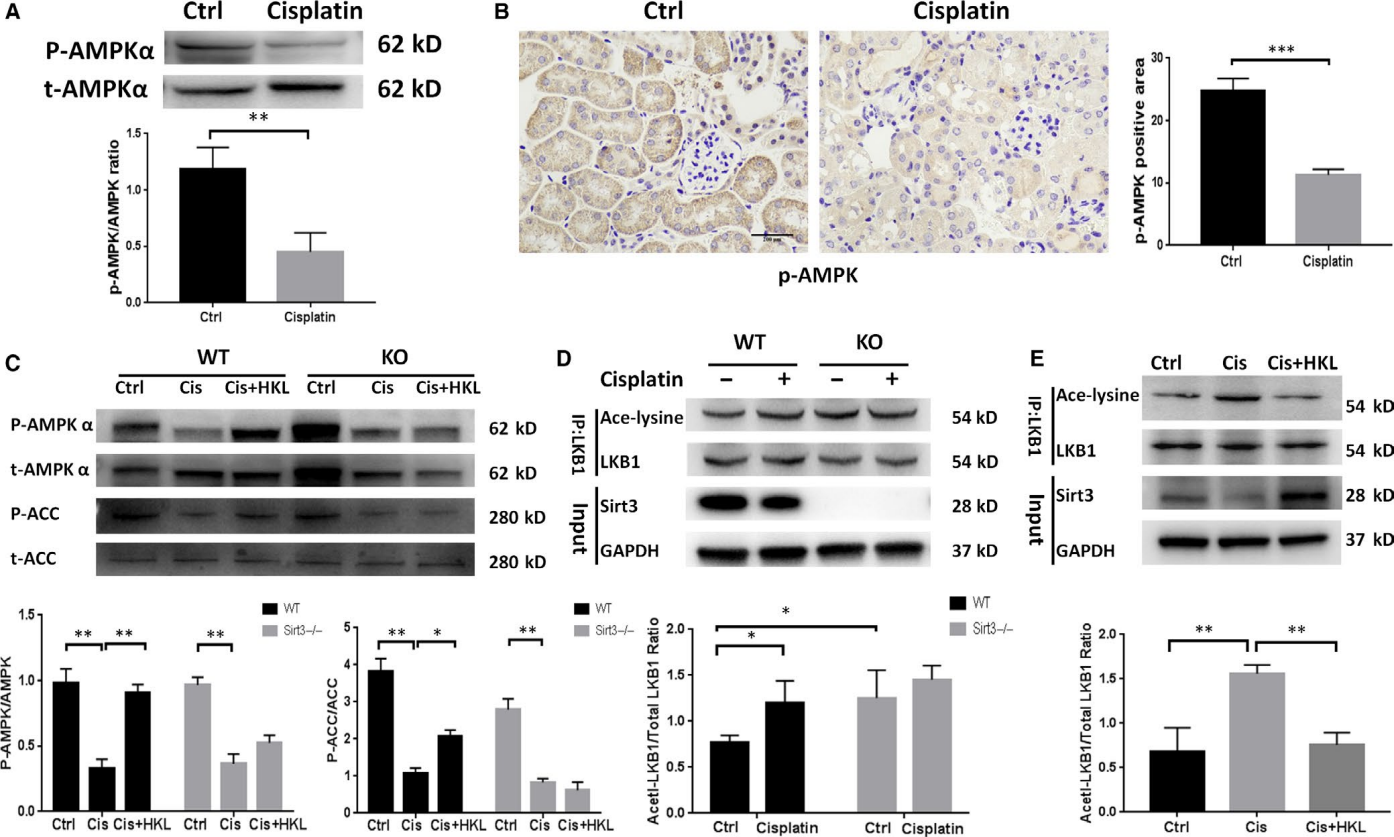
Sirt3 may regulate FAO through the LKB1‐AMPK pathway. A, Western blot and densitometric analysis of P‐AMPKα and total AMPKα in the control and cisplatin groups of mRTECs, **P < .01, n = 3. B, Representative phospho‐AMPKα immunohistochemical staining of mouse kidney sections and semiquantitative positive scoring of phospho‐AMPKα staining among different groups, scale bar, 100 µm. C, Western blot and densitometric analysis of P‐AMPKα/total AMPKα and P‐ACC/total ACC in the kidneys of each group, *P < .05, **P < .01, n = 3. D, Immunoprecipitation and densitometric analysis of acetylated LKB1 in each group of mice, *P < .05, n = 3. E, Immunoprecipitation and densitometric analysis of acetylated LKB1 in vitro, **P < .01, n = 3

To further delineate the pathway by which Sirt3 regulates FAO following cisplatin treatment, we investigated whether the activation of AMP‐activated protein kinase (AMPK) contributes to FAO. In vitro, we first observed the changes of AMPK activity, which documented as the ratio of phosphorylated AMPKα (pAMPKα) to AMPKα. Cisplatin‐treated mRTECs exhibited considerably lower levels of AMPK activity compared to those in control mRTECs (Figure 5A). This is consistent with the result previously described by Morigi et al.^12^ The expression of phosphorylated AMPKα was also decreased in AKI kidney tissue by immunohistochemical staining (Figure 5B). ACC (acetyl‐CoA carboxy), which converts acetyl‐CoA to malonyl‐CoA, an inhibitor of CPT1, is the rate‐limiting enzyme involved in FFA synthesis and the downstream effector of AMPKα. In WT mice with AKI, pre‐treatment with HKL increased the phosphorylation of AMPKα and ACC compared to those treated with cisplatin alone. However, these effects were not observed in Sirt3 KO mice (Figure 5C). These results indicate that HKL may up‐regulate the phosphorylation of AMPKα and ACC by activating Sirt3. To further clarify how Sirt3 promotes the phosphorylation of AMPKα, we examined the effect of Sirt3 on liver kinase B1 (LKB1), which is the upstream activator of AMPK.^30^ Sirt3 is a protease with deacetylation function, and it has been reported that LKB1 was inactivated by acetylation.^31^ Therefore, we hypothesize that Sirt3 may activate LKB1 through deacetylation and then promote the phosphorylation of AMPK. Immunoprecipitation was used to test the hypothesis. LKB1 acetylation was substantially increased in KO mice compared to that in WT mice (Figure 5D), suggesting that Sirt3 was capable of deacetylating LKB1 in the kidney with AKI. In addition, the same results were observed in cultured cells as well. After the up‐regulation of Sirt3 expression in tubular epithelial cells by HKL, the acetylated LKB1 level was significantly lower than that in cells treated with cisplatin alone (Figure 5E).

### 3.6 Sirt3 activation attenuates cisplatin‐induced mitochondrial dysfunction and energy utilization disorder

**Figure 6:**
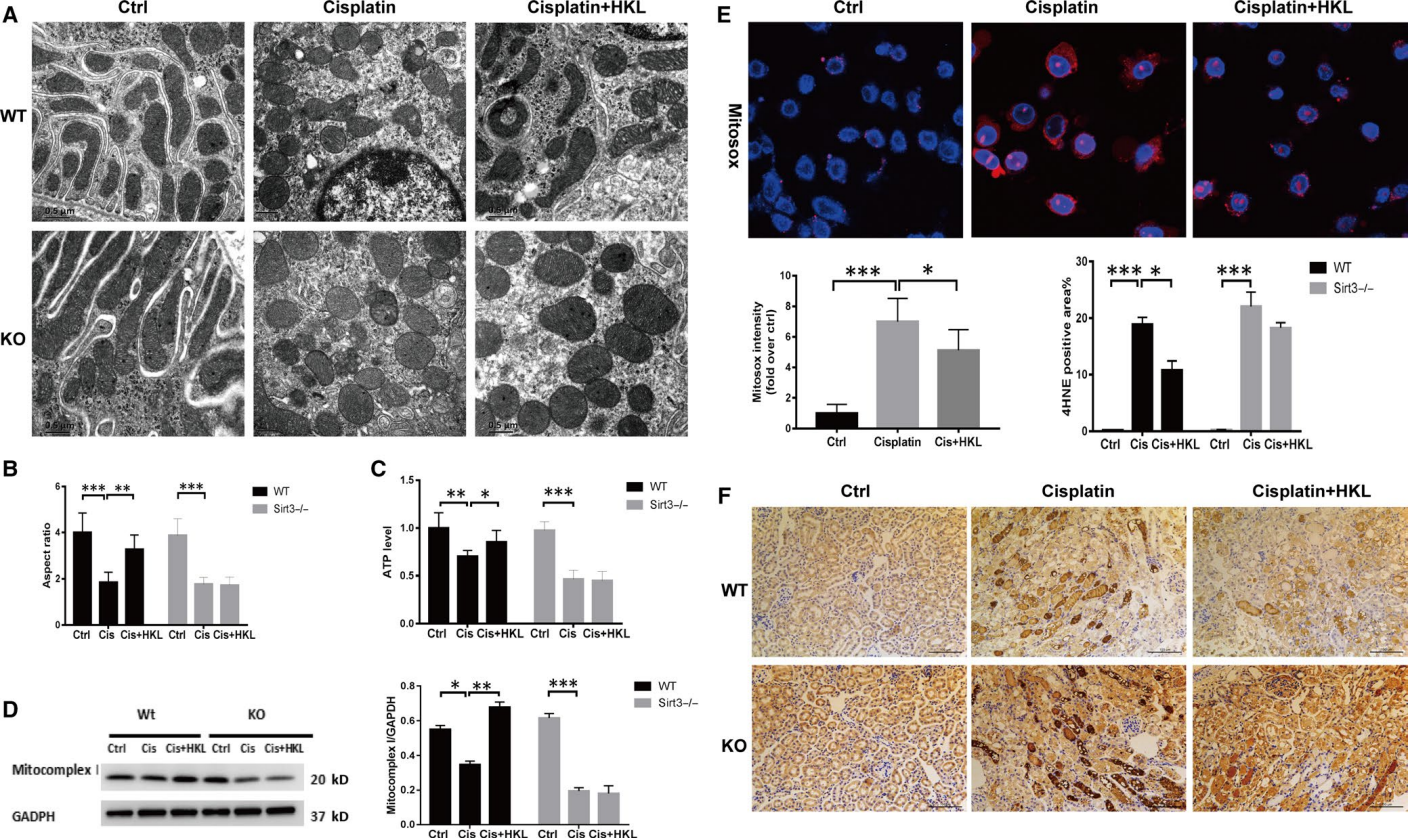
Sirt3 activation attenuates cisplatin‐induced mitochondrial damage and lipid peroxidation. A, Transmission electron microscopy showed the morphological mitochondrial structure in renal tubular epithelial cells from each group. Scale bar, 0.5 µm. B, Quantitative comparison of the transverse width of each group of mice. **P < .01, ***P < .001, n = 20. C, Comparison in the ATP levels between the groups of mice, *P < .05, **P < .01, ***P < .001, n = 5; D, Western blot and densitometric analysis of the mitochondrial complex I in each group, *P < .05, **P < .01, ***P < .001, n = 3. E, Representative confocal images and quantitative analysis of mitochondrial ROS detected by MitoSOX, scale bar, 10 µm. F, Representative 4HNE immunohistochemical staining of mouse kidney sections and semiquantitative positive 4HNE staining scores among the different groups, scale bar, 100 µm

As FAO is closely related to mitochondrial function, we examined the role of Sirt3 in it. First, we examined whether Sirt3 prevented cisplatin‐induced mitochondrial morphological damage. As shown by TEM (Figure 6A,B), small, fragmented, swollen and abnormal mitochondrial profiles and disrupted cristae were evident in the kidneys of cisplatin‐treated WT mice. However, these morphological changes were attenuated in HKL‐pre‐treated mice. In KO mice with AKI, these morphological changes were worse, and HKL preconditioning did not improve the mitochondrial profile. We next examined whether Sirt3 activation improved ATP production, which was found to be lower in KO AKI mice than that in WT AKI mice. After HKL treatment, ATP production increased significantly in WT AKI mice but not in KO AKI mice (Figure 6C). Similar results were also observed for respiratory chain‐related protein mitochondrial complex I expression (Figure 6D). The production of mitochondrial ROS in mRTECs that were treated with HKL + cisplatin was also observed to be significantly lower than in mRTECs that were treated with cisplatin alone (Figure 6E). We further observed the effect of activating Sirt3 on lipid peroxidation in vivo. The expression of 4HNE in kidney tissues from KO AKI mice was more significant than that in WT AKI mice, and after pre‐treatment with HKL, the expression of 4HNE in WT AKI mice decreased significantly, but not in KO AKI mice (Figure 6F).

## 4 DISCUSSION

In this study, we found extensive fatty acid deposition in cisplatin‐induced AKI mice that was closely related to cisplatin‐induced fatty acid oxidative damage and mitochondrial dysfunction. FAO dysfunction leads to decreased ATP synthesis in tubules and lipoapoptosis, which aggravates AKI. Sirt3 plays an important role in FAO regulation and mitochondrial function. In addition to the previously known mechanisms of Sirt3,^14^, ^32^ we found that Sirt3 further regulated FAO via deacetylation of LKB1.

The kidney is a huge energy‐consuming organ. Correspondingly, a large number of mitochondria are distributed in renal tubular epithelial cells to synthesize energy, and the mitochondrial density is only second to that of myocardial cells.^33^ Current studies have confirmed that fatty acid oxidation is the most important fuel in proximal tubular cells,^10^, ^34^ and FAO defects and mitochondrial dysfunction are universally involved in diverse causes of AKI. The ATP produced by 1 mol of palmitic acid is three times greater than that produced by 1 mol of glucose.^35^ Under ischaemic and toxic conditions, mitochondrial damage and FAO dysfunction causes reduced ATP production and inhibited sodium‐potassium‐ATPase activity, which results in a sodium influx that is 3‐4 times greater than normal and induces cell swelling.^36^ Therefore, mitochondrial damage and FAO dysfunction are early initiating factors of AKI. Our study confirmed that mice with cisplatin‐induced AKI had significant FAO dysfunction and fatty acid deposition, which is consistent with previous reports.^7^, ^8^, ^9^, ^37^, ^38^ Mass spectrometry analysis of metabolites indicated that fatty acid oxidative damage resulted in insufficient energy substrates for the downstream TCA cycle and reduced ATP production. The latter can impede actin aggregation; further, cytoskeleton breakdown causes brush border loss and cell detachment in RTECs.^34^, ^36^, ^39^ Meanwhile, intracellular ATP levels have been implicated as a determinant of apoptosis, as ATP is needed for activation of the caspase cascade.^40^ FAO also serves as a critical mediator of cell death in AKI not only by reducing ATP generation, but also because of FFA‐related lipotoxicity, which represented as mitochondrial dysfunction associated with an increase in ROS production and ATP production loss, apoptosis and elevated inflammatory cytokines.^41^, ^42^ Injured mitochondria become a robust source of excess ROS, and ROS are prone to react rapidly with FFA, resulting in the formation of lipid peroxides such as HNE, and induce lipid apoptosis. Here, we confirmed the possibility of lipid apoptosis in cisplatin‐induced AKI mice in vitro and in vivo. And activation of Sirt3 by HKL can reduce ROS production and lipid peroxidation.

According to the literature and our previous studies,^12^, ^13^, ^25^ Sirt3 may play an important role in regulating mitochondrial function via modulation of the fission and fusion processes. Sirtuin3 is mainly located in mitochondria and exhibits global mitochondrial lysine deacetylase activity.^11^ Recent studies have shown that the use of human mesenchymal stem cells in mice with AKI can accelerate the regeneration of renal tubular epithelial cells. The main mechanism of this effect is the improvement of mitochondrial function in mesenchymal stem cells through the Sirt3 pathway.^43^ Absence of Sirt3 aggravates cisplatin nephrotoxicity via enhanced renal tubular apoptosis and inflammation.^44^ Sirt3 also acts as an antioxidant stress by deacetylating SOD2 and P53.^45^ In addition, in an AKI model induced by sepsis, researchers also observed that Sirt3 plays a protective role against mitochondrial damage in the kidney by attenuating ROS production, inhibiting the NRLP3 inflammasome, attenuating oxidative stress and down‐regulating pro‐inflammatory cytokines.^46^ Sirt3 has been reported to regulate LCAD and ACSS2 through deacetylation during the regulation of fatty acid metabolism.^13^, ^47^ However, whether Sirt3 regulates FAO in AKI and improves the prognosis of AKI has not yet been reported. Our study showed that Sirt3 participates in the regulation of FAO in cisplatin‐induced AKI. When Sirt3 was knocked out, FAO damage was aggravated; when Sirt3 was up‐regulated, FAO disorder was alleviated, and renal function was improved. We further investigated whether SIRT3 modulates the expression of PPARα, CPT1 and ACADL, which are the key molecules in regulating FAO.^8^, ^34^, ^48^, ^49^ In cisplatin‐mediated AKI, PPARα activation induces expression of its target's genes such as CPT‐1 or ACADL, thereby reducing lipid accumulation in kidney tubular.^42^, ^49^ In the present study, we found that in AKI mice, deletion of Sirt3 reduced the expression of PPARα, while HKL pre‐treatment restored it, which indicates that Sirt3 can regulate FAO by modulating PPARα expression. As of now, the relationship between Sirt3 and PPARα remains unclear. Recently, a study reported that Sirt3, as the upstream of PPARα, regulates the expression of PPARα to affect autophagy.^50^ But how Sirt3 affects PPARα expression remains to be further explored.

To further elucidate the pathway of FAO, which is regulated by Sirt3, we focused on the AMPK pathway. AMPK is an important energy receptor in cells. When ATP level decreases and the AMP level increases, the ratio of AMP to ATP increases, which activates the AMPK pathway.^51^ AMPK acts on a series of downstream, such as ACC, PPAR and PGC1α, resulting in increased mitochondrial biosynthesis, glycolysis and fatty acid oxidation, and thus increases cellular energy and promotes cell growth.^52^ We observed that AMPK phosphorylation decreased after treatment with cisplatin in mRTECs. Similar results were also observed in vivo, with a decrease in ACC phosphorylation and consequently increased ACC activity. The latter decreases CPT1 activity and inhibits FAO via the malonyl‐CoA pathway.^53^ Moreover, HKL pre‐treatment did not improve AMPK and ACC phosphorylation levels in Sirt3 KO mice, suggesting that Sirt3 may increase AMPK phosphorylation and activate AMPK and downstream pathways. Similar to our results, it was reported that Sirt3 overexpression can protect hepatocytes against lipotoxicity by activating AMPK.^54^ Palacios et al^55^ also reported that phosphorylated AMPK and PGC1α were down‐regulated in Sirt3 knockout mice compared with their levels in WT mice. However, in AKI mice, how Sirt3 regulates AMPK remains unclear. It has been reported that in hepatocytes, honokiol activated the LKB1‐AMPK signalling pathway and attenuated the lipid accumulation.^56^ Pillai et al^57^ also revealed that Sirt3 could activate LKB1 by deacetylating LKB1 in mice with myocardial hypertrophy and then regulate AMPK phosphorylation to alleviate myocardial hypertrophy. In a review, Ansari et al summarized the ability of Sirt3 to deacetylate LKB1.^58^ Inspired by these studies, and considering that Sirt3 is an important deacetylase, we focused on the role of LKB1 as a bridge between Sirt3 and AMPK. We first examined the expression of acetylated LKB1 in mice with cisplatin‐treated AKI and found that LKB1 acetylation was evident in AKI mice. We further observed the acetylation of LKB1 after Sirt3 knockout. A high degree of LKB1 acetylation occurred in Sirt3 KO mice in both the control and cisplatin treatment group. These results indicate that Sirt3 may regulate fatty acid oxidation by LKB1 acetylation and thus regulate the AMPK signalling pathway.

As known, cisplatin has direct renal toxicity. Many studies have demonstrated that cisplatin can drive mitochondrial damage and that Sirt3 has a protective effect on the mitochondria. In this study, we also found that the activation of Sirt3 can significantly improve mitochondrial morphology and functions, increase ATP production and reduce ROS and lipid peroxidation. However, it remains difficult to elucidate whether the role of Sirt3 in improving FAO is independent of mitochondrial function or whether it is a beneficial result of its mitochondrial protection, as they interact with each other. As the mitochondria are the site for FAO, the down‐regulation of FAO enzymes may be closely related to damaged mitochondrial morphology and function.^34^, ^35^ However, the accumulation of fatty acids in AKI can also create a toxic environment inside cells, which hinders mitochondrial function.^34^, ^59^ Some studies have shown that, in VLCAD‐deficient mice, the accumulation of long‐chain fatty acids and carnitine derivatives can disrupt mitochondrial homeostasis, which includes the uncoupling of OXPHOS, reduction of mitochondrial membrane potential, and induction of apoptosis and necrosis.^60^, ^61^ Based on the present study, we speculate that Sirt3 attenuates both pathological processes. Sirt3 could improve FAO by both protecting mitochondrial function and restoring the expression and activity of key enzymes. As such, improved FAO reduces lipotoxic substances and ROS, which thus maintains mitochondrial homoeostasis.

Increasing evidence suggests that FAO dysfunction and mitochondrial impairment are important links in the progression of kidney disease. In addition to its role in AKI, FAO dysfunction is also an important mechanism for the progression of CKD.^10^ Our data demonstrate that restoring FAO may provide a new therapeutic approach for the treatment and prevention of AKI, in which Sirt3 plays an important role. However, the underlying mechanism remains to be further explored. Therefore, the development of novel drugs targeting FAO and the mitochondrion are expected to become an important focus in the future.

## CONFLICTS OF INTEREST

The authors declare no conflicts of interest.

## AUTHOR CONTRIBUTIONS

Tanqi Lou, Hui Peng and Ming Li conceived and designed the experiments. Ming Li, Canming Li, Zengchun Ye, Jiayan Huang, Yin Li and Weiyan Lai performed the experiments. Ming Li wrote the paper. Ming Li, Hui Peng and Canming Li reviewed/edited the manuscript. All authors read and approved the final version of the manuscript.

## Supporting information


**Figure S1**

## Data Availability

The data that support the findings of this study are available from the corresponding author upon reasonable request.
