# Supplementary figures and images for: Sirt3 modulates fatty acid oxidation and attenuates cisplatin‐induced AKI in mice
Source: J Cell Mol Med. 2020 Apr 12;24(9):5109–21. doi: 10.1111/jcmm.15148 (PMC7205836; doi:10.1111/jcmm.15148)

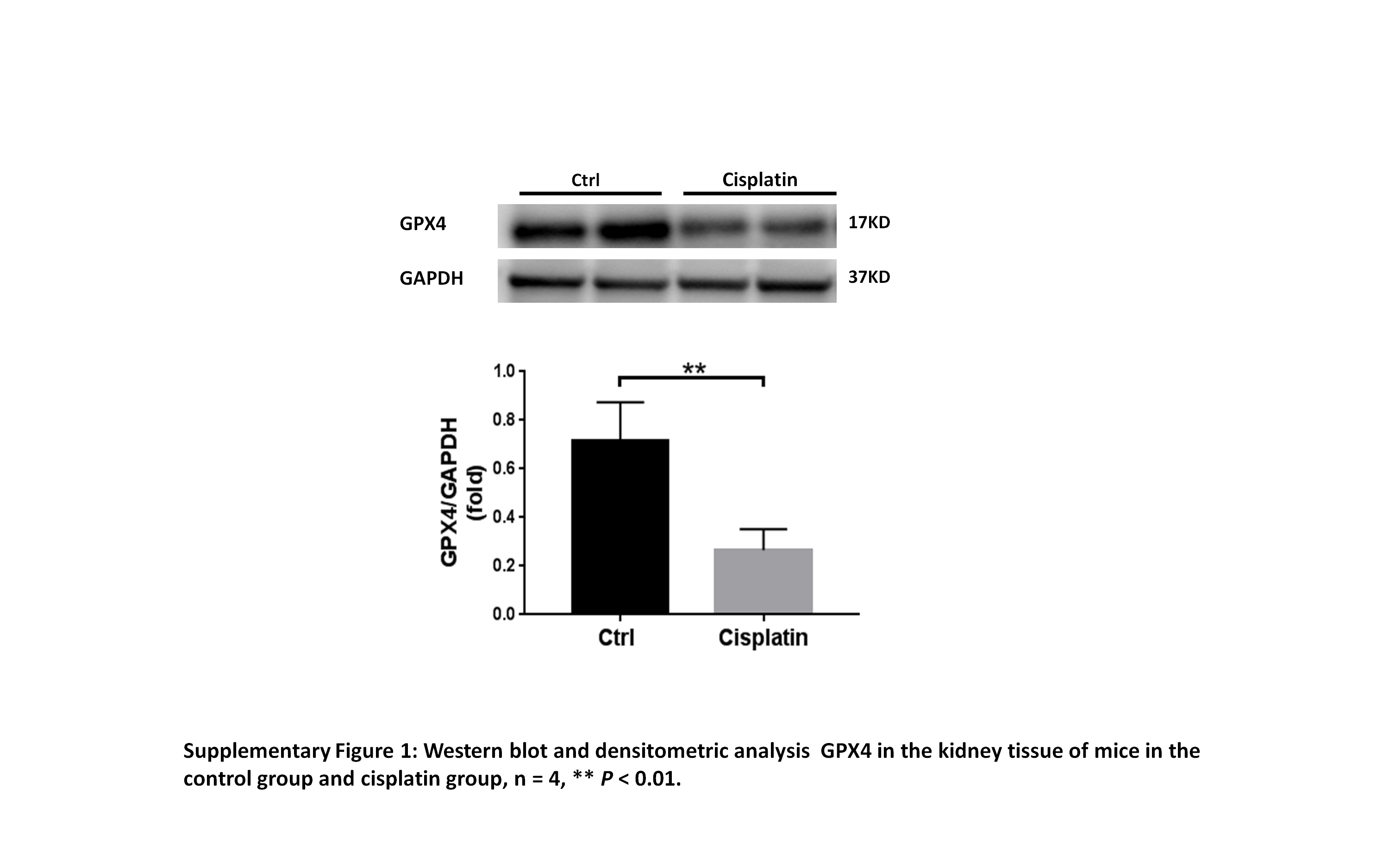

Supplement: Supplementary file 1 — Figure S1 [file JCMM-24-5109-s001.png]
